# Digital planning of composite customized veneers using Digital Smile Design: Evaluation of its accuracy and manufacturing

**DOI:** 10.1002/cre2.570

**Published:** 2022-04-01

**Authors:** Luca Ortensi, Giancarlo Sigari, Giusy Rita Maria La Rosa, Agnese Ferri, Francesco Grande, Eugenio Pedullà

**Affiliations:** ^1^ Department of General Surgery and Medical‐Surgical Specialties University of Catania Catania Italy; ^2^ Oral and Maxillofacial Surgery University of Bologna Bologna Italy; ^3^ Department of Prosthodontics University of Ferrara Ferrara Italy

**Keywords:** esthetic restoration, composite veneers, digital preview, Digital Smile System

## Abstract

**Objectives:**

This study aimed to evaluate the production of customized composite veneers starting from a two‐dimensional (2D) digital preview using the Digital Smile System (DSS).

**Material and Methods:**

A photographic examination of 30 patients was performed by taking two digital pictures of the face and a digital preview through the DSS. Moreover, optical scans of the dental arches were obtained and the data were entered into a three‐dimensional (3D) software to prepare a virtual preview. The standard tessellation language files were sent for production using CAD‐CAM technology. The Friedman test, Bonferroni, and Dunn post hoc tests were used, comparing the linear measurements of the 2D and 3D plans and the final veneers (*α* = .05).

**Results:**

Significant differences emerged between the pictures and digital scans on the mesial–distal widths of the lateral incisors and canine. Linear measurements in the 2D plan were significantly different from those of the 3D plan, except for the height measures of incisors. No significant changes were found on comparing the parameters of the 2D and 3D plans with those of the final pieces.

**Conclusions:**

The customized veneers were clinically adequate and similar to 2D and 3D plans, although significant differences emerged between the picture and digital scans as well as between the 2D and 3D plans.

## 1 INTRODUCTION

In recent decades, dental medicine has undergone rapid changes due to the development of various branches; among these, restorative dentistry has progressed considerably from a clinical and research perspective (Watson et al., 2013). Dental technologies have increased along with patient expectations. In particular, for patients, the esthetic result has become as important as the functional recovery. The benchmarks of modern dentistry are utmost respect for healthy tooth tissues and, even more, conservative dental preparations. “Bioeconomy,” or “minimal invasiveness,” and “biomimetics” are easily achievable goals by using composite and ceramic materials, as well as the enamel–dental adhesive techniques currently available in esthetic dentistry (Farias‐Neto et al., 2019).

In recent years, the increased production of indirect composite restorations has been associated with improved properties of the materials. New composite resins have properties that fulfill most practical needs, for instance, adequate strength and monomer curing, color stability, high wear resistance and polishability retention, and superior esthetic and handling characteristics (Ferracane, 2011). Because of the development of these new resins and the use of modern adhesive techniques, currently, composite veneers can be considered as valid therapeutic alternatives for the treatment of esthetic abnormalities affecting the anterior teeth, as well as ceramic materials (Pini et al., 2012). Composite veneers offer some advantages compared with ceramic restorations such as lower intrinsic fragility, reduced cost, and easy manageability in the event of eventual fractures and subsequent repairs (Van Meerbeek et al., 2010). These reasons and the need for customized veneers that satisfy the patient's expectations make the composite one of the best materials for esthetic restorations (Watson et al., 2013). The indications for their use can be subdivided into three groups: dental discoloring resistant to whitening procedures; need for significant morphological changes of anterior teeth; and extensive restoration of compromised anterior teeth (Watson et al., 2013). The alternative solution to veneers is the production of direct composite restorations, which requires minimal preparation of the tooth; however, they are susceptible to wear, micro infiltration, and fractures (Dudea et al., 2015). Conversely, the indirect composite veneer technique is an optimal treatment option to avoid these issues due to its greater resistance (D'Arcangelo et al., 2014; Gresnigt et al., 2019).

To date, professionals choose the best treatment in agreement with the patient, achieving the so‐called “therapeutic compromise” (Ortensi, Lo Castro, et al., 2020). Furthermore, use of two‐dimensional (2D) and three‐dimensional (3D) software, associated with digital image editing, enables processing of the images, including any clinical–esthetic specification, and reliable virtual simulations (Virtual Planning) that facilitate dialogue between the patient and the dental technician (Cervino et al., 2019). After taking a series of pictures of the face and intraoral area, the clinician, based on the previously described esthetic principles (Cervino et al., 2019), makes changes to dental elements on obtaining a preview of the treatment (Ortensi et al., 2019). The DSS (Digital Smile System Srl, Bologna, Italy) is one of the most commonly used virtual planning systems (Jokstad, 2017). This software enables digital planning of esthetic and functioning rehabilitation of the smile (Sanchez‐Lara et al., 2019), offering a preview of the final result, available both to the patient and to all members of the therapeutic group.

To the best of our knowledge, no studies have investigated the possibility to produce a prototype or prosthetic element that has the same measurements as those previewed using 2D software. Therefore, the aim of this study was to assess the production of customized composite veneers that conformed as best as possible to the digital preview and to the patients' esthetic demands. The null hypothesis was that no difference would be found in the measurements of the 2D and the 3D plans, as well as of the final customized piece.

## 2 MATERIAL AND METHODS

Thirty patients who spontaneously presented to our observation from a dental private practice in Bologna, from September 2019 to February 2020, requesting restoration treatment to improve the esthetics of maxillary anterior section (from canine to canine), were enrolled in this study. All patients signed an informed consent. The patient sample included 18 women and 12 men between 20 and 50 years of age, none of whom presented ideal esthetic proportions of dental elements, as described by many authors (Belser, 1982; Magne et al., 2003; Richard, 1973; Spear et al., 2006).

Inclusion criteria were as follows: good oral hygiene, periodontal health, and adult patients requiring esthetic/functional restorations of the maxillary anterior region (canine to canine). Exclusion criteria were as follows: history of orthodontic treatment, restoration/cavities, missing teeth in the maxillary anterior region, severe bruxism or clenching, and misalignments and periodontal defects in the maxillary anterior region. Following the Virtual Planning Guidelines, the position of the central maxillary incisors was assessed as a starting point for rehabilitation of the smile (Fradeani & Barducci, 2008).

The system used for this study relies on a phonetic test to detect the proper position of the maxillary incisors, in particular observing labial movement during pronunciation of the M and I phonemes (Calamita et al., 2019; D'Arcangelo et al., 2018).

### 2.1 Photographic protocol

**Figure 1:**
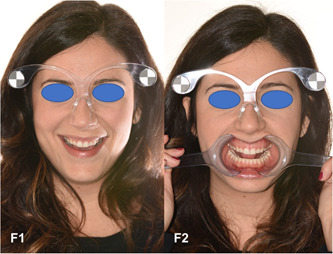
(F1) Full face picture with spreaders showing the arches semi‐disclosed. (F2) Full face picture with a wide smile showing the arches semi‐disclosed

After occlusal, phonetic, static, and dynamic assessments of each patient, two digital photographs of the face were taken, with the eyes, ears, and lips visible. To keep patients in a position that was stable and repeatable over time, they were seated upright so that the Frankfurt Plane (the anatomical plane joining the porion and orbital points) was parallel to the horizon. The camera was positioned at the same height of the patient's face, with vertical modality, to be able to look directly into the lens, and the bipupilar plane was as parallel as possible to the horizontal plane. The first photograph (F1) of the face was taken with spreaders, with slightly disclosed dental arches to correctly assess both the parallelism between the bi‐pupillary and occlusal planes and the congruence of the median and interincisive lines. The second photograph (F2) of the face was taken after removing the spreaders and with the patients smiling, exposing their teeth to assess the alignment of the incisive plane compared with the lower lip, as well as the width of the lateral corridors (Ortensi, Lo Castro, et al., 2020; Ortensi, Vitali, et al., 2020) (Figure 1).

To conduct the photographic protocol, a digital single‐lens reflex (DSLR) camera (D300; Nikon Corporation, Tokyo, Japan) was used with the EX Sigma 105 mm 1:2.8 DG macro lens with a 6.5 diaphragm opening. The Nikon SB‐R 200 flash system was used, equipped with a LumiQuest pocket bouncer, mounted on a Medical Close‐up Scorpion bracket (Nikon Corporation).

The photographic support was mounted onto a 120‐cm tripod at a distance of 150 cm from the patient for both photographs (Isa et al., 2010).

For the pictures of faces, the participants wore dedicated glasses (Figure 1) with an optical measurement system that enables conversion of pixels into millimeters through photographic markers (Ortensi, Lo Castro, et al., 2020; Ortensi, Vitali, et al., 2020). This method enabled the clinician to take the photos in a repeatable way. Moreover, this procedure ensured the perpendicular position between the patient and the camera for the virtual process.

### 2.2 Digital planning

**Figure 2:**
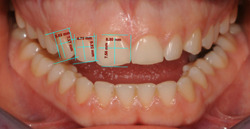
Linear measurements of frontal area elements

After the photographic examination, the dental arches were subjected to optical scanning (Trios, 3Shape A/S, Copenaghen, Denmark). The digital photographs were imported into the 2D DSS (Digital Smile System Srl) for the virtual planning of the veneers of the maxillary anterior section including the identifying data and the patient's consent to process them. Using the digital callipers integrated in the software, the *Guideline* screen displayed the linear measurements of the maxillary right central and lateral incisors, and canine from the incisive edge (for incisors) or the tip cusp (for canines) to the gingival zenith and mesial–distal widths at the equator (Figure 2).

**Figure 3:**
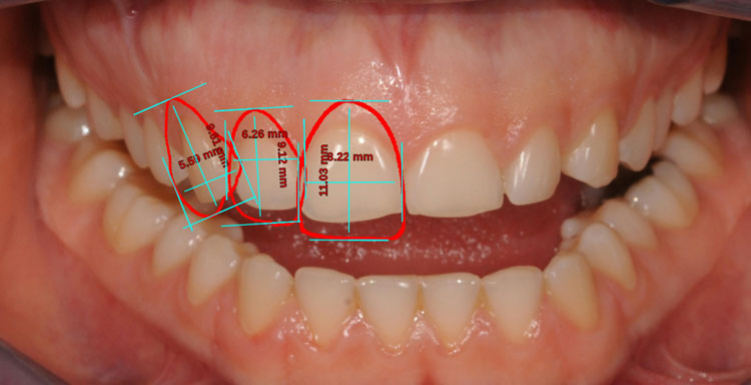
Measurements of the frontal area performed from the outlines

**Figure 4:**
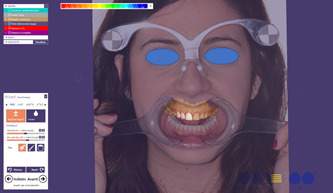
The transition of the 2D plan to Exocad is supported by the alignment phases of the outlines of the 2D plan to the digital scan of the patients' arches. The 3D modeling phase was then performed through transformation of the 2D library into an STL (standard tessellation language) file

Following the dental virtual planning, the same linear measurements were performed on the *Dental Positioning* screen for the 2D plan (Figure 3). Then, the PDF report was extracted for each project. Afterwards, the optical impressions of dental arches were overlapped with the F1 pictures of the patients and processed using 3D Exocad® DentalCAD 2.2 Valletta software (Exocad GmbH), referring to the 2D outlines obtained by processing the chosen library (Sanchez‐Lara et al., 2019) (Figure 4).

The standard tessellation language (STL) files obtained from processing the 3D plan were sent for the production of composite veneers using computer‐aided design and computer‐aided manufacturing (CAD‐CAM) subtractive milling technology (New Ancorvis Srl, Bologna, Italy) and dedicated composite disks (Brilliant Crios HT; Coltene/Whaledent, Altstatten, Switzerland).

### 2.3 Data analysis

**Figure 5:**
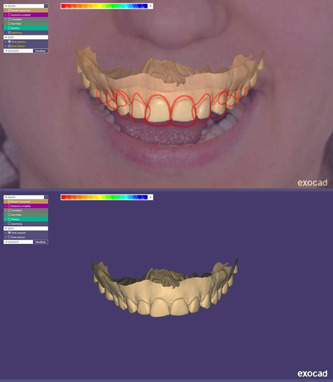
Pairing of picture F1 to the arch scan allowed us to set the patient's upper arch in the same position as that of the photographs, enabling easier comparison of the measurements taken on the 2D images

Subsequently, the linear measurements of the maxillary right central and lateral incisors and canine were replicated from the incisive edge (for incisors) or the tip cusp (for canines) to the gingival zenith and mesial–distal widths at the equator on the patient arch scanning and the scan screenshots after pairing with F1 in order to fix the position in space and to use the same reference points for the linear measurements (Figure 5). This process was performed using 3Shape 3D Viewer® software (3Shape A/S). Afterwards, the same measurements were performed on the composite veneers using the Digital Calliper (0−150 mm, Mitutoyo, Japan) and on the 3D plan using Exocad software (Exocad GmbH). Once these measurements were performed, the veneers were scanned (SinergiaScan; NobilMetal SpA, VillaFranca D'Asti, Italy) and overlapped with the 3D plan to assess the consistency level of the individual customized pieces.

**Figure 6:**
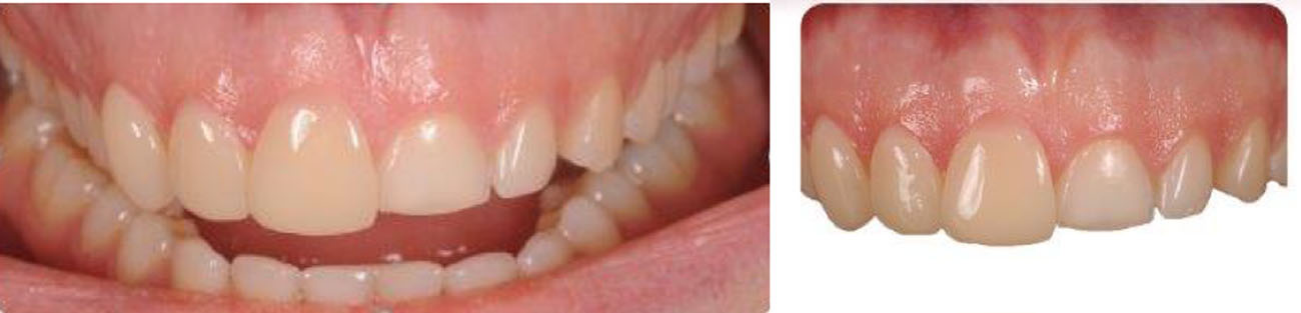
Veneers of elements (maxillary right central incisor—maxillary right lateral incisor—maxillary right canine) were tested in the patients' mouths

**Figure 7:**
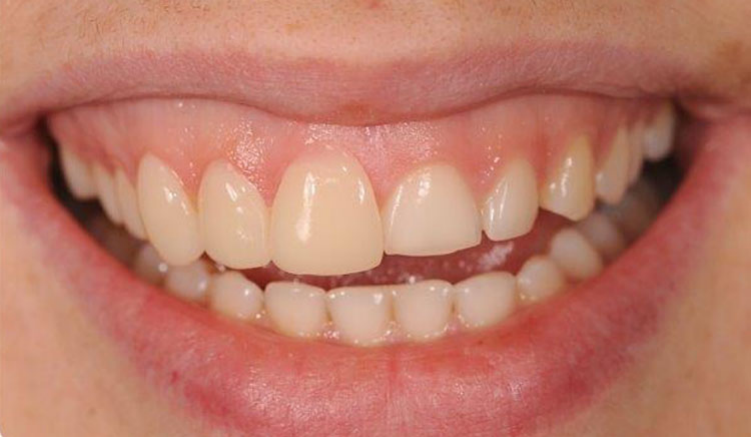
Phonetic testing performed in the oral cavity

Once these procedures were completed, the veneers were provisionally fixed with a nonpolymerized composite and tested in the oral cavity of the participants (Fahl & Ritter, 2021). The prosthetic fit was evaluated through initial phonetic and occlusal tests for each patient (Figures 6 and 7). Afterwards, the consistency between the 2D virtual preview of the treatment and the pieces made was assessed, replicating the prior detection phase of the two standard F1 and F2 pictures, after the placement of the veneers. The data were then imported into 2D DSS software for the linear measurement of the maxillary right central and lateral incisors and canine as well as the mesial–distal widths of the restorations, following the protocol. All measurements were performed independently by two operators.

### 2.4 Statistical analysis

Interexaminer agreement was measured using the Cohen kappa (*κ*) values. Any differences among the measurements of the two operators were resolved by consensus. The measurement data were statistically analyzed using a software program (Prism 8.0; GraphPad Software, San Diego, CA, USA). Normality of the data was determined using the Shapiro–Wilk test, and the Friedman test for matched groups with Bonferroni and Dunn tests for multiple comparisons were used (*α* = .05).

## 3 RESULTS

The *κ *value for interexaminer reliability was 0.85 for 2D measurements, 0.83 for 3D measurements, and 0.81 for customized composite veneers.

### 3.1 2D project accuracy

The means and standard deviation (SD) of the linear measurements of maxillary right central and lateral incisors, and canine before the virtual planning are shown in Table 1.

**Table 1:** The means and standard deviation (SD) of the linear measurements of the maxillary right central and lateral incisors and canine before the virtual planning

	Maxillary right central incisor (mean ± SD)		Maxillary right lateral incisor (mean ± SD)		Maxillary right canine (mean ± SD)	
	*H*	MD	*H*	MD	*H*	MD
PICTURE	8.43 ± 0.62^a^	8.05 ± 0.66^a^	6.95 ± 0.79^a^	5.48 ± 0.36^a^	8.12 ± 1.01^a^	4.72 ± 0.42^a^
3D ARCH SCAN	8.27 ± 0.44^a^	8.05 ± 0.65^a^	6.80 ± 0.61^a^	6.12 ± 0.58^b^	7.84 ± 0.54^a^	7.05 ± 0.33^b^
2D ARCH SCAN SCREENSHOT	8.18 ± 0.39^a^	7.78 ± 0.57^a^	6.63 ± 0.52^a^	5.28 ± 0.42^a^	7.73 ± 0.57^a^	4.52 ± 0.61^a^

*Note*: Superscript letters indicate a statistically significant difference (*p* < .05).

Abbreviations: 2D ARCH SCAN SCREENSHOT, screenshot of upper arch scans (after pairing with F1) measured with 3Shape 3D Viewer®; 3D ARCH SCAN, scans of the upper jaw measured with 3Shape 3D Viewer®; DSS, Digital Smile System; *H*, height; MD, mesial−distal; PICTURE, F1 pictures measured with DSS.

The heights measured from the pictures showed no significant difference compared with those measured from the digital scans (*p* > .05). However, a significant increase was found in the mesial–distal widths of the lateral incisor and canine (*p* < .05). These discrepancies were not found in the comparison of the same widths measured on the pictures against those of the screenshot of upper arch scans after pairing with F1 (*p* > .05).

### 3.2 3D project accuracy

The means and standard deviation of the measurements of the 2D plan (DSS), the 3D plan (3Shape 3D Viewer®), and the customized piece (digital calliper) are shown in Table 2.

**Table 2:** The means and standard deviation (SD) of the measurements of the 2D plan (DSS), the 3D plan (3Shape 3D Viewer®), and the customized piece (digital calliper)

	Maxillary right central incisor (mean ± SD)		Maxillary right lateral incisor (mean ± SD)		Maxillary right canine (mean ± SD)	
	*H*	MD	*H*	MD	*H*	MD
2D PLAN	10.56 ± 0.49^a^	8.47 ± 0.17^a^	8.94 ± 0.55^a^	6.19 ± 0.37^a^	9.78 ± 0.57^a^	5.75 ± 0.77^a^
3D PLAN	10.67 ± 0.65^a^	8.91 ± 0.51^b^	9.12 ± 0.55^a^	6.88 ± 0.42^b^	10.13 ± 0.56^b^	7.84 ± 0.30^b^
DIGITAL CALLIPER	10.69 ± 0.63^a^	8.99 ± 0.54^ab^	9.19 ± 0.58^a^	7.01 ± 0.39^ab^	10.14 ± 0.58^ab^	7.94 ± 0.34^ab^
SCREENSHOT 3D PLAN	10.24 ± 0.44^a^	8.38 ± 0.33^a^	8.88 ± 0.45^a^	6.25 ± 0.25^a^	9.76 ± 0.44^a^	5.83 ± 0.52^a^

*Note*: Superscript letters indicate a statistically significant difference (*p* < .05).

Abbreviations: DSS, Digital Smile System; *H*, height; MD, mesial−distal.

In this case, by comparing the mesial–distal widths as well as heights of all teeth, a significant increase was found in the step from the 2D plan to the 3D plan (*p* < .05), except for height measures of the right central and lateral incisors, for which no significant difference was found between 2D and 3D plans (*p* > .05). For the 2D plan, another comparison was performed with the 3D plan screenshots after pairing with the intraoral pictures along the line of the arch scans, and no significant differences emerged (*p* > .05).

Once prosthetic manufactures were completed, the linear measurements on 2D and 3D plans were compared with those of the composite veneers obtained by a digital calliper, and no significant differences in the heights and mesial−distal widths emerged (*p* > .05).

## 4 DISCUSSION

The aim of this study was to evaluate the accuracy of digital planning for customized composite veneers. According to the International Organization for Standardization (ISO), “accuracy” refers to the combination of trueness and precision. The former is linked to the closeness of a measure to the exact value of the size. The latter is related to how close several measurements of the same quantity are to each other (ISO 5725‐1:1994, 1994). Although previous studies confirmed the accuracy and the reduced procedural errors of the digital workflow for porcelain veneers manufacturing (Gürel et al., 2012; Reshad et al., 2008), to the best of our knowledge, this is the first study to verify the accuracy of digital panning on composite veneers. Composite customized veneers represent an acceptable alternative to ceramic ones due to their valid esthetic result combined with reduced costs and the possibility for easy repair (Pini et al., 2012; Van Meerbeek et al., 2010). Use of a digital caliper is a standardized and validated method for the measurement of teeth because of “its acceptable accuracy, practicality, portability, and lower cost” (Liu et al., 2021). In addition, in the present study, all 2D and 3D measurements were performed independently by two operators. The Cohen *κ* values used to verify the interexaminer agreement confirmed the reliability and reproducibility of the procedure.

Based on the results obtained, the null hypothesis was rejected. No significant differences were found between the heights measured from the pictures compared with those measured from the digital scans, except for the mesial–distal widths of the lateral incisors and canine, in which a significant increase was found in digital scans. It is possible to hypothesize that this discrepancy is due to the major inclination of the dental elements involved. Indeed, because of the 2D nature of photos, the mesial–distal widths tend to be distorted from the central incisors toward the posterior sectors (Lavorgna et al., 2019). Once 2D photos (i.e., F1 and F2 images) were paired with the digital scans, screenshots were taken to compare the new 2D images with the initial pictures. At this stage, no significant discrepancies were identified. This result confirmed that the differences found between pictures and digital scans in mesial–distal widths were due to the passage from the 2D environment to the 3D one. The same trend can be observed for the 2D and 3D plans: a significant increase was recorded for the mesial–distal widths of all dental elements and for the height of maxillary right canine from the 2D to 3D. These results were obtained probably due to the difficulty in standardizing the measurements made from the different dental inclinations in 2D and 3D plans. Differences in 2D and 3D projects are plausible because they are performed in different environments (i.e., 2D and 3D) and thus a perfect match between them is not ensured. In addition, measurements performed on DSS are clinically relevant because DSS represents the tool used by clinicians in their routine. Conversely, Exocad measures are less practical and informative for the operators due to the expense and complexity of the 3D Exocad software, which reduce its application in clinical practice.

An additional comparison between the 2D and 3D virtual plans with the composite customized veneers was performed, yielding no significant differences. These results showed the clinical adequacy of the customized veneers, which were clinically tested, showing satisfactory prosthetic fit. Clinical adequacy refers to a good clinical fit intended by stable engagement and no significant clinical adaptations required (Lo Giudice et al., 2020).

Our aim was to confirm that the final customized product was overlapping with the initial project, viewed and approved by the patient. The results obtained are clinically relevant because they confirm that the final composite customized veneers are comparable with the 3D plans and conform to the esthetic preview provided to the patient. Nevertheless, the reduced sample size and the type of material and software tested are possible limitations. Further studies on a larger sample of participants and alternative materials and digital systems are necessary to confirm these results.

## 5 CONCLUSIONS

Within the limitations of this study, the evaluation of the accuracy of digital planning for composite veneers manufacturing showed that:

(1) the linear measurements on the pictures were similar to those of the digital scans, except for the mesial–distal width of the lateral incisors and canine, in which a significant increase was found in digital scan measures;
(2) significant differences were found between the linear measurements on the 2D plan compared with the 3D one, particularly on mesial–distal widths; and
(3) the final composite customized veneers were comparable with the 2D and 3D plans, confirming the clinical adequacy of the final prosthetic pieces.

## AUTHOR CONTRIBUTIONS

*Conceptualization*: Luca Ortensi and Eugenio Pedullà. *Data curation*: Luca Ortensi, Eugenio Pedullà, and Giusy Rita Maria La Rosa. *Formal analysis*: Giancarlo Sigari, Agnese Ferri, and Giusy Rita Maria La Rosa. *Investigation*: Giancarlo Sigari and Francesco Grande. *Methodology*: Luca Ortensi, Eugenio Pedullà, and Agnese Ferri. *Project administration*: Luca Ortensi. *Resources*: Luca Ortensi and Eugenio Pedullà. *Software*: Luca Ortensi, Giancarlo Sigari, and Agnese Ferri. *Supervision*: Luca Ortensi and Francesco Grande. *Validation*: Luca Ortensi, Eugenio Pedullà, and Francesco Grande. *Visualization*: Agnese Ferri and Francesco Grande. *Roles/Writing*—*original draft*: Giancarlo Sigari and Giusy Rita Maria La Rosa. *Writing*—*review & editing*: Luca Ortensi, Eugenio Pedullà, and Giusy Rita Maria La Rosa.

## CONFLICTS OF INTEREST

The authors declare no conflicts of interest.

## ETHICS STATEMENT

All procedures performed in studies involving human participants were in accordance with the ethical standards of the institutional research committee and with the 1964 Helsinki Declaration. The Research Ethics Committee has confirmed that no ethical approval is required. Informed consent was obtained from all individual participants included in the study. The authors affirm that human research participants provided informed consent for publication of the images in Figures 1 and 4.

## Data Availability

Data sharing not applicable—no new data were generated.
